# Supplementary material for: Seminoma and Embryonal Carcinoma Footprints Identified by Analysis of Integrated Genome-Wide Epigenetic and Expression Profiles of Germ Cell Cancer Cell Lines
Source: PLoS One. 2014 Jun 2;9(6):e98330. doi: 10.1371/journal.pone.0098330 (PMC4041891; doi:10.1371/journal.pone.0098330)
Supplement: File S1 — ZIP file containing DMRforPairs output for significant regions. Please start from the html files. (ZIP) [file pone.0098330.s008.zip › figures/30231.pdf]

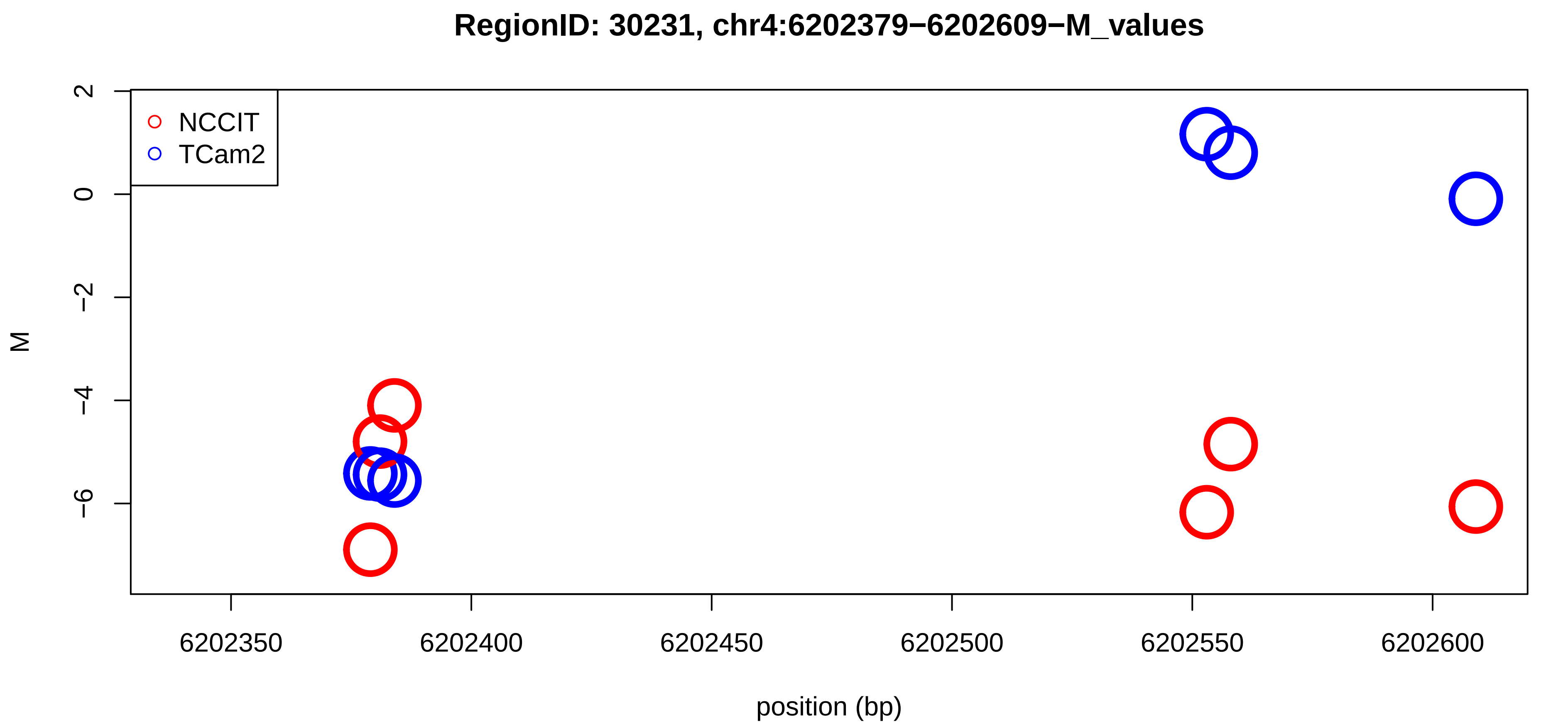

RegionID: 30231, chr4:6202379−6202609−M_values
M
2
0
−2
−4
−6
NCCIT
TCam2
6202350
6202400
6202450
6202500
6202550
6202600
position (bp)

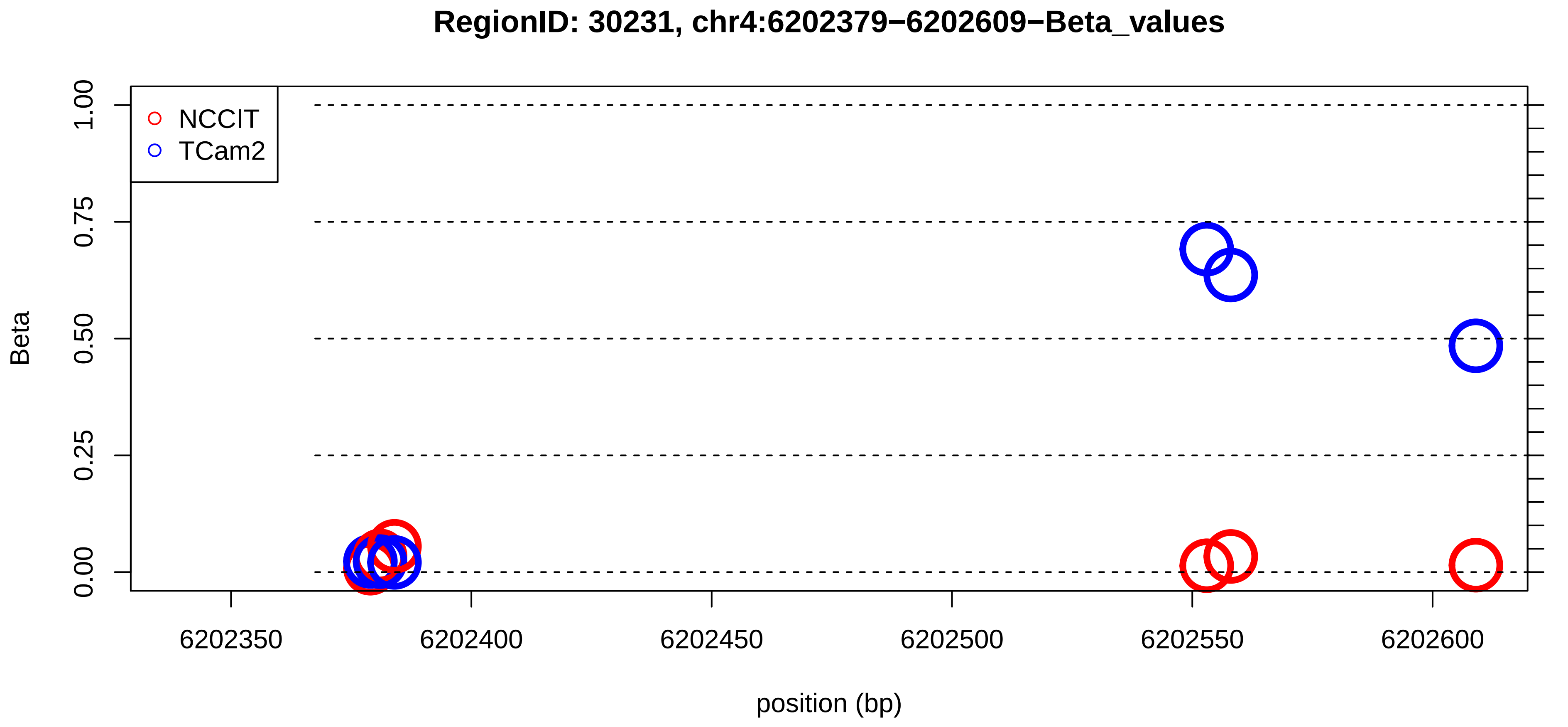

RegionID: 30231, chr4:6202379−6202609−Beta_values
Beta
0.00
0.25
0.50
0.75
1.00
NCCIT
TCam2
6202350
6202400
6202450
6202500
6202550
6202600
position (bp)
